# Deepfake smiles matter less—the psychological and neural impact of presumed AI-generated faces

**DOI:** 10.1038/s41598-023-42802-x

**Published:** 2023-09-26

**Authors:** Anna Eiserbeck, Martin Maier, Julia Baum, Rasha Abdel Rahman

**Affiliations:** 1 Department of Psychology, Faculty of Life Sciences, Humboldt-Universität zu Berlin, Unter den Linden 6, 10099 Berlin, Germany; 2 Cluster of Excellence Science of Intelligence, Technische Universität Berlin, Berlin, Germany

**Keywords:** Perception, Emotion, Human behaviour, Social behaviour

## Abstract

High-quality AI-generated portraits (“deepfakes”) are becoming increasingly prevalent. Understanding the responses they evoke in perceivers is crucial in assessing their societal implications. Here we investigate the impact of the belief that depicted persons are real or deepfakes on psychological and neural measures of human face perception. Using EEG, we tracked participants’ (N = 30) brain responses to real faces showing positive, neutral, and negative expressions, after being informed that they are either real or fake. Smiling faces marked as fake appeared less positive, as reflected in expression ratings, and induced slower evaluations. Whereas presumed real smiles elicited canonical emotion effects with differences relative to neutral faces in the P1 and N170 components (markers of early visual perception) and in the EPN component (indicative of reflexive emotional processing), presumed deepfake smiles showed none of these effects. Additionally, only smiles presumed as fake showed enhanced LPP activity compared to neutral faces, suggesting more effortful evaluation. Negative expressions induced typical emotion effects, whether considered real or fake. Our findings demonstrate a dampening effect on perceptual, emotional, and evaluative processing of presumed deepfake smiles, but not angry expressions, adding new specificity to the debate on the societal impact of AI-generated content.

As generative artificial intelligence is becoming more widely available, computer-generated images and videos of people that appear deceptively real (“deepfakes”), continue to move into our daily lives^1–4^. The social impact of these technologies and recommendations for their ethical application depend substantially on psychological factors within the perceivers^5^. How do people deal with the fact that they are increasingly confronted with faces that may or may not be real? To date, the psychological and neural consequences of knowing or suspecting an image to be a deepfake remain largely unknown. When real and fake faces are otherwise indistinguishable, perception and emotional responses may crucially depend on the prior belief that what you are seeing is in fact real or fake. To illustrate, consider AI-generated art: recently, a song written by ChatGPT "in the style of Nick Cave" left the artist himself deeply unimpressed: "with all the love and respect in the world, this song is […] a grotesque mockery of what it is to be human. […] Songs arise out of [human] suffering"^6^. This implies that a song acquires aesthetic and emotional relevance when we *know* that an actual human being created it from real experience.

Likewise, perceivers’ beliefs can be an important factor for the impact of deepfake images, whether in beneficial or potentially harmful applications. For instance, images produced by generative adversarial networks (GAN) serve as realistic anonymous stock photos in research, advertising or personal profiles^7,8^, or bring back younger versions of movie characters. Here, GAN images are intended to evoke the same perceptual and emotional responses as seeing a real person, even when recipients know that they aren’t real. On the other hand, knowledge about the mere existence of deceptively real fakes can sow distrust and uncertainty about the credibility of information^9^. Misinformation campaigns, such as discrediting reports as “fake news”, also rely on the assumption that the belief of seeing a fake will lead people to discount the content. In addition, the effect of believing an image to be real or fake may depend on the emotional valence of the content. Negative person-related information was shown to influence social impressions and associated brain responses, whether it came from trustworthy or untrustworthy sources. Positive information, in contrast, is sometimes discounted when perceived as untrustworthy^10,11^.

Recent rating studies demonstrated the challenges faced by human observers in discerning between GAN faces and real face images^2,11^. However, the neurocognitive mechanisms underlying this phenomenon and the impact of presumed deepfakes on face perception and evaluation remain poorly understood. Here, we investigate these mechanisms drawing upon insights from psychology and cognitive neuroscience, which offer viable paradigms to study the influence of context and prior beliefs on perception^13–15^. For instance, people interpret facial expressions using information from contextual body postures^16^, or read emotional expressions into objectively neutral faces based on their beliefs about the character of the depicted person, even if those beliefs are based on untrustworthy information^10,11,17,18^. Brain responses have revealed effects of such prior beliefs and context on different processing stages, including early perception, reflexive emotional processing, and higher-level evaluation^10,11,13,17–21^.

In the current study, we used EEG to track participants’ brain responses to faces displaying positive, neutral, and negative expressions (smiles, neutral, and angry faces) with high temporal resolution, after being informed that they are either real or GAN faces. Because we were interested in the effects of beliefs about fake- vs. realness, and to exclude otherwise possible confounds due to low-level physical differences of the presented images, all faces shown in the study were actually of existing people. For each face, participants rated how positive or negative they perceived the facial expression. Do the facial expressions of deepfakes matter as much as real ones?

We used event-related potentials (ERPs) to isolate systematic patterns of brain activity in response to the appearance of face images on a screen. A set of well-described ERP components can reveal the consequences of expecting deepfake vs. real images for different processes along the information processing stream, from lower-level visual perception to emotional processing and higher-level evaluation. To test effects on early visual face perception, we looked at the P1 and N170 components. The P1 peaks around 100 ms after stimulus presentation and reflects low-level processing, such as perceived contrast^22^, and the N170, peaking around 170 ms, has been reported to be particularly sensitive to the processing of faces and facial expressions^23,24^. Do facial expressions actually *look* different when we assume that they come from deepfakes? To study reflexive emotional processing, we analyzed the early posterior negativity (EPN)^25–27^: are smiles and angry faces as emotionally relevant when they presumably come from deepfakes? Finally, we looked at the late positive potential (LPP), an index of sustained evaluative processing of emotionally relevant stimuli^10,27^. Do presumed deepfakes make our brains “pause and look twice” when evaluating facial expressions?

We preregistered the following predictions (https://osf.io/xymz8). Concerning a potential main effect of information (deepfake vs. real), we predicted the processing of presumed deepfake faces to be characterized by reduced activity in the N170, EPN and LPP components^28,29^. Concerning the interaction of information and emotion displayed by the face, we predicted the perceived intensity of facial expressions, as measured in valence ratings, and canonical effects of emotional facial expressions on the N170, EPN, and LPP to be reduced for presumed deepfakes. We also predicted that this modulation may be stronger or restricted to happy faces, i.e., indicating an especially pronounced discounting of fake smiles, or likewise, a prioritization of threatening faces no matter their status as real or fake^11,30^.

## Methods

### Participants

The sample comprised 30 participants (21 female, 9 male) with a mean age of 25.87 (standard deviation [*SD*] = 4.98) years. Participants provided written informed consent before participation. The study was conducted according to the principles expressed in the Declaration of Helsinki and was approved by the ethics committee of the Department of Psychology at Humboldt-Universität zu Berlin. Participants received either course credit or monetary compensation of €10 per hour.

Initial datasets of 7 participants (4 female, 3 male; mean age *M* = 26, *SD* = 6.06) were excluded and replaced, keeping the predefined sample size of 30 participants and ensuring a balanced within-subject design. Datasets were excluded based on preregistered criteria. These included cases where participants answered less than 7 out of 12 control questions during the free viewing task correctly, indicating a lack of attention during the task (2 participants), and where the number of remaining observations per individual cell (information × emotion condition) was less than 30 out of 60 after the removal of ERP trials containing artifacts (2 participants). Further aspects that affected data quality, such as excessive blinking during stimulus presentation (2 participants) and misunderstanding of the instructions (1 participant), were also considered.

The sample size was based on a priori power estimations as well as sample sizes of previous studies investigating the effects of verbal information on face perception^10,17,18^. Power estimations were run on simulated EPN data with the SIMR package^31^ in R. We based the analysis on a main effect of Information, expecting a difference in mean EPN amplitude of at least 0.3 µV between faces presented as “real” and those presented as “fake.” A linear mixed model was specified to predict mean EPN amplitude by Information (Real / Fake), including by-participant intercepts. We aimed for a power of at least 80% as conventionally deemed adequate^31^. After running 1,000 randomizations given different sample sizes, results indicated that testing at least 18 participants would yield an expected power of 85.30%, 95% CI [82.95, 87.44] to detect the predicted effect. In order to have sufficient power to detect potential interaction effects between Information (Real, Fake) and Emotion (Angry, Neutral, Happy) as well as condition-specific effects, and taking into account the balanced experimental design requiring a multiple of 6 participants, we ultimately chose to test 30 participants.

### Materials

Pictures of 180 different human faces (90 female, 90 male) with a Caucasian appearance, each with three emotional expressions (angry, neutral, happy), served as stimuli, amounting to a total of 540 pictures. The photographs were obtained from three databases: The “Radboud Faces Database” (RaFD)^32^, “Karolinska Directed Emotional Faces” (KDEF)^33^, and “FACES”^34^. The face stimuli were cropped into an oval shape, excluding hair, ears, and neck. Contrary to what was stated in the cover story (see procedure), all pictures were real photographs, i.e., no artificially created pictures were included in this study.

Information about the real or (alleged) artificial nature of the faces was given via the written words “REAL” or “FAKE.” In the experiment, for each participant, 50% of faces were labeled as “REAL” and 50% as “FAKE.” Each participant saw each one of the 180 face identities in only one emotion condition (Angry/Neutral/Happy) and one information (Real/Fake) condition. The assignment of conditions was counterbalanced across participants, such that each face was shown equally often in all six conditions, i.e., with all three expressions shown in both information conditions.

Stimuli were presented on a 19-inch LCD monitor with a 75-Hz refresh rate on a gray background. The faces were displayed with a size subtending 9.59° vertical and 6.81° horizontal visual angles (viewing distance: 70 cm).

### Procedure

After participants provided informed consent, the EEG was prepared. Subsequently, the experimenter read a standardized introduction explaining GAN faces while a video showing possibilities in creating GAN faces was played on the computer screen without audio (see Supplement S1 for verbatim instructions and video information). It was highlighted that nowadays, it is possible to create artificial faces that are often indistinguishable from real faces. At the end of the introduction, participants were informed that in the experiment, they would see such artificial as well as real faces and that they would be informed about this via the preceding word “REAL” or “FAKE.” Following the instructions, participants had the chance to clarify any remaining questions. Afterward, the experimenter left the EEG cabin, and the experiment started.

The experiment consisted of two parts: a free viewing task and a facial expression rating task, which were always carried out in this exact order. The present manuscript focuses on the rating task, which provided behavioral data (facial expression ratings and reaction times) as well as ERPs following face presentation, enabling an examination of effects on both the behavioral and neurophysiological levels. Analogous analyses of ERPs in the free viewing task can be found in Supplement S4.

The rating task consisted of 2 blocks. Within each block, all 180 face stimuli were displayed once in randomized order, together with the accompanying information (“REAL”/”FAKE”), yielding a total of 360 rating trials, with 60 trials per individual Emotion × Information condition. Short pauses were included after every 30 trials.

**Figure 1:**
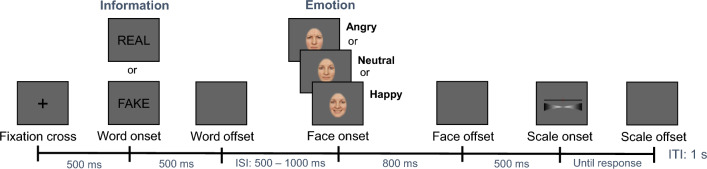
Overview of the time course of one experimental trial during the rating phase. Each trial started with a fixation cross, followed by the information (“REAL” or “FAKE”), and the face stimulus, which displayed one of three emotions (angry, neutral, or happy expression). Afterwards, participants rated the valence of the facial expression on a continuous 100-point scale, with endpoints labeled as “very negative” and “very positive”. ISI = Inter-stimulus interval; ITI = Inter-trial interval.

Figure 1 provides an overview of the trial structure of the rating task. Each trial started with the presentation of a fixation cross in the center of the screen for 500 ms. Afterward, either the word “REAL” or “FAKE” was displayed for 500 ms in the same location. Then a blank screen was presented for a random duration between 500 and 1000 ms before face stimulus onset. Therefore, the shortest possible duration between word and face onset amounted to 1000 ms, and the longest possible duration was 1500 ms. Subsequently, the respective face stimulus was displayed for 800 ms. Afterward, a rating scale for facial expression judgments was displayed 500 ms after the face offset (1300 ms after face onset). The continuous rating scale contained 100 points (values: 1–101), with the endpoints labeled as “very negative” and “very positive” (direction of the scale counterbalanced across participants). Participants had been instructed on the use of the scale before starting the task. By moving a slider on the scale, they chose the point that best represented their judgment of the valence of the expression. The scale was presented until the participant chose a value on the scale via mouse click. Then, after an inter-trial interval of 1 s, the next trial started. The obtained rating values and response times served as dependent variables for behavioral data analyses.

The free viewing task, which preceded the rating task, contained the same number of trials and followed the same structure, except for the following differences: Trials during the free viewing task already ended with face offset. This part contained no task except for occasional attention checks to ensure that participants paid attention to the information: A control question was shown at a random point in time once within every 30 trials, asking whether the face presented last was “real” or “fake” (according to the given information). The participant answered via button press. A total of 12 control questions were shown (only) during the free viewing task.

After the experiment, participants performed a short task to collect eye movements for artifact correction and filled in a questionnaire about demographic information and the experiment (assumptions about hypotheses; open comments). Finally, they were debriefed that the experiment did not actually contain AI-generated faces.

### EEG recording and preprocessing

The EEG was recorded with Ag/AgCl electrodes at 64 scalp sites according to the extended 10–20 system at a sampling rate of 500 Hz and with all electrodes referenced to the left mastoid. An external electrode below the left eye was used to measure electrooculgrams. During recording, low- and high-cut-off filters (0.016 Hz and 1,000 Hz) were applied, and all electrode impedances were kept below 10 kΩ. After the experiment, a calibration procedure was used to obtain prototypical eye movements for later artifact correction. Processing and analyses of the data were based on the EEG processing pipeline by^35^. Offline preprocessing was conducted using Matlab (R2018b, MathWorks Inc.) and the EEGlab toolbox^36^. The continuous EEG was re-referenced to a common average reference, and eye movement artifacts were removed using a spatio-temporal dipole modeling procedure with the BESA software^37^. The corrected data were low-pass filtered with an upper passband edge at 40 Hz. Subsequently, they were segmented into epochs of −200 to 1000 ms relative to face stimulus onset and baseline-corrected using the 200 ms pre-stimulus interval. Segments containing artifacts (amplitudes over ± 150 µV, changing by more than 50 µV between samples) were excluded from further analysis. For statistical analyses, data were exported to R (Version 4.2.2)^38^. Single-trial mean amplitudes were obtained for the P1, N170, EPN, and LPP components by averaging across time ranges and electrode sites typical for the respective component, specified as follows:

P1: The analyses included parieto-occipital electrode sites (O1, O2, Oz, PO7, PO8). The time range for analysis was determined by peak detection for data collapsed across all conditions (“collapsed localizer”)^39^ within the predefined time range from 80 to 130 ms^28,40^. The peak detection yielded a mean P1 peak at 118 ms (SD = 12.38). A range of approximately 2 SD before and after the mean peak was selected, yielding a time range of 90-140 ms for analyses.

N170: The analyses included parieto-occipital electrode sites (TP9, TP10, P7, P8, PO9, PO10, O1, O2). The analysis time range was determined as described for the P1, with a predefined time range for peak detection from 130 to 200 ms^41^. The peak detection yielded a mean N170 peak at 166.7 ms (SD = 12.78). A range of approximately 2 SD before and after the mean peak was selected, yielding a time range of 140–190 ms for analyses.

EPN: The analyses included posterior electrodes (PO7, PO8, PO9, PO10, TP9, TP10) and a time range from 200 to 350 ms^17,18,42^.

LPP: The analyses included centro-parietal electrodes (Pz, Cz, C1, C2, CP1, CP2) and a time range from 400 to 600 ms^17,42^.

### Analyses

Statistical analyses were conducted using linear mixed-effects models^43^ as implemented in the lmer function of the lme4 package^44^ for R. The lmerTest package^45^ was utilized to calculate p-values.

For all models, we used the buildmer package^46^ to specify the random effects structure. It implements an automatic procedure to identify the maximal random effects structure with which the model still converges and to perform backward stepwise elimination to avoid overfitting (for further details on buildmer specifications, see Supplement S2).

Single-trial mean amplitudes for the P1, N170, EPN, and LPP components served as dependent variables (in separate models) in the ERP analyses. For each dependent variable (i.e., each ERP component, behavioral ratings, response times), the following model was specified (each model also including random effects as described above): The model included the fixed factors information (real/fake), emotion (angry/neutral/happy) and their interaction. Effect coding was applied for the factor information (0.5, −0.5). Repeated contrast coding (also called sliding difference) was applied for the factor emotion to compare the levels angry and neutral, as well as happy and neutral separately^47^. Hence, the model coefficients were: the main effect of information, the main effect of negative emotion (angry vs. neutral), the main effect of positive emotion (happy vs. neutral), the interaction of information and negative emotion, and the interaction of information and positive emotion. We tested the significance of fixed effects coefficients (*p*-value < 0.05) by Satterthwaite approximation (summary function of lmerTest).

Planned contrasts were calculated from the model to test emotion effects within each information condition separately and to test possible differential influences of information on the happy condition vs. the angry condition. For this purpose, we employed the emmeans package^48^.

### Additional exploratory analyses: Response times

In addition to the pre-registered analyses, we analyzed response times during the facial expression task. Response times were measured from scale onset until response (mouse click on the scale). Response time data were trimmed using the sdTrim function of the trimr package^49^ such that responses faster than 200 ms and slower than 2.5 SD above the mean per Emotion × Information condition for each participant were excluded from analyses (mean number of excluded trials per participant = 13.7, SD = 5.84). To ensure a normal distribution of residuals for linear mixed model analyses and based on a Lambda value close to 0 in the boxcox plot, response time data were log-transformed. The analysis was based on log-transformed response times as the dependent variable, and the same model specifications as described above were used.

## Results

**Figure 2:**
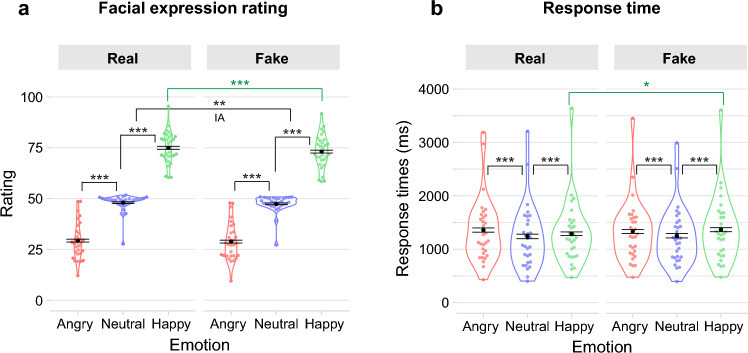
Facial expression ratings (**a**) and response times (**b**) depending on Information and Emotion conditions. Violin plots are based on by-participant means per condition. Black points show condition means and error bars represent 95% confidence intervals with inter-subject variance removed. Correspondence of rating values in (**a**): 1 = very negative, 51 = neutral, 101 = very positive. Asterisks indicate significant differences between the respective conditions and a significant interaction between Information and Positive Emotion (labeled as “IA” in the plot) observed in the analyses: *** *p* < .001, ** p < .01, * *p* < .05. An alternative illustration showing by-participant differences between Real and Fake conditions per Emotion is provided in Supplement Fig. S5.

**Figure 3:**
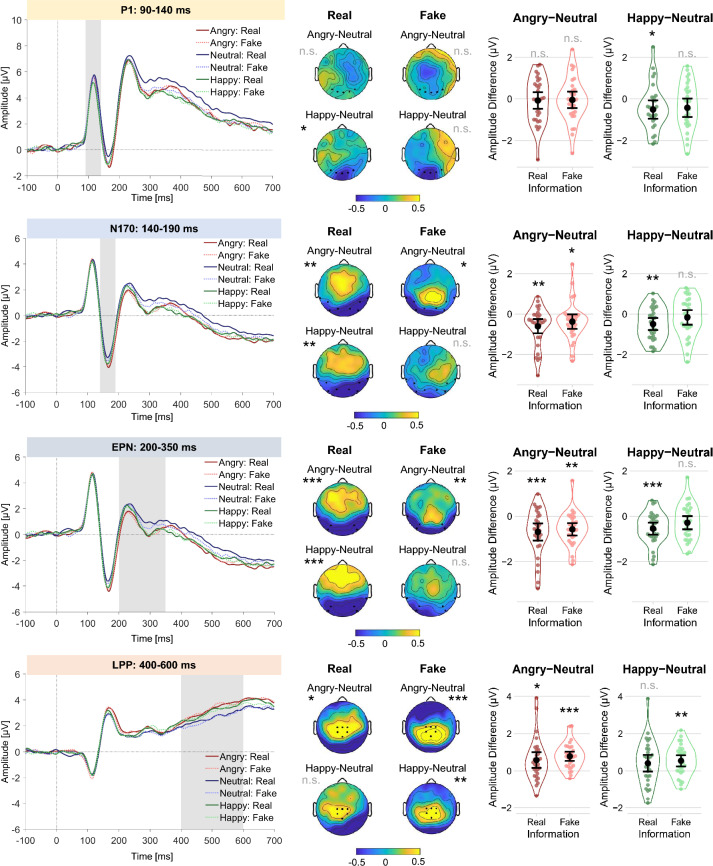
Differences in activity of the P1, N170, EPN, and LPP components depending on Information (Real / Fake) and Emotion (Angry / Neutral / Happy) during the rating task. Left: Grand-average amplitudes following face onset, pooled across the respective region of interest (ROI) for each component. The gray shading marks the time range taken into account for the analyses for each component. Middle: For each component, topographies show the differences in activity between Angry and Neutral, and Happy and Neutral faces for each Information condition. Asterisks indicate a significant difference between the respective conditions observed in the analyses. Right: Amplitude differences between Angry and Neutral, and Happy and Neutral faces for both Information conditions. The colored dots represent by-participant differences of the respective conditions; the black dot represents the mean difference; error bars display 95% confidence intervals around the mean. Asterisks indicate significant differences between the respective conditions observed in the analyses. *** *p* < .001, ** p < .01, * *p* < .05; n.s. = non-significant.

**Figure 4:**
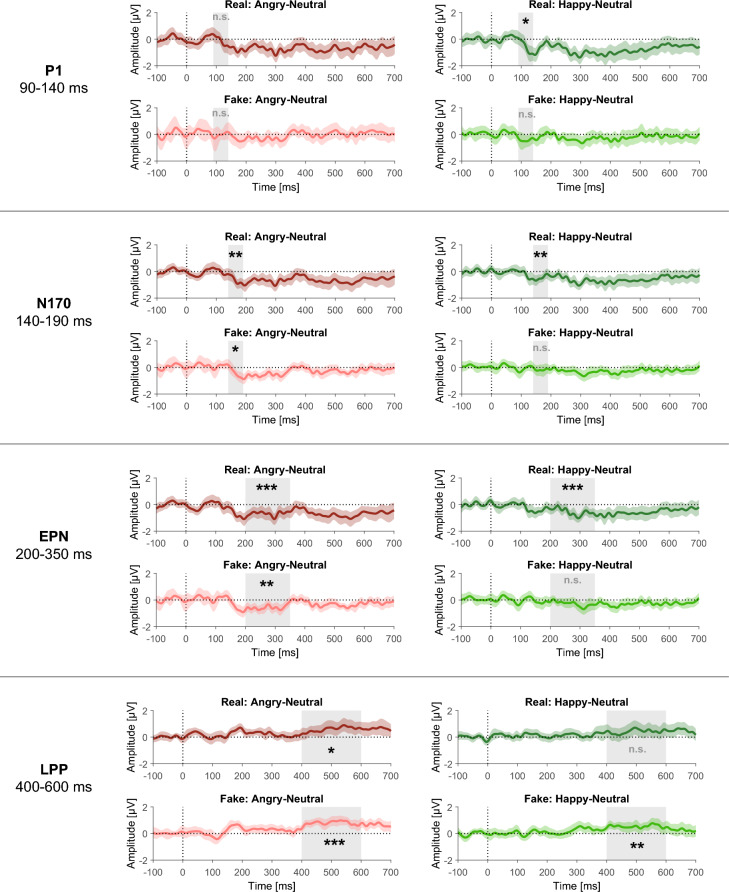
Differential ERPs time-locked to face onset display the differences between the Angry and Neutral conditions, and between the Happy and Neutral conditions within each Information condition (Real/Fake) for the tested components. For each component, the differences between conditions based on pooled activity across electrodes included in the respective region of interest (ROI) are shown. Error bands represent 95% confidence intervals around the difference, obtained via bootstrapping. The gray shading marks the time range taken into account for the analyses for each component. Asterisks indicate significant differences between the respective conditions observed in the analyses. *** *p* < .001, ** p < .01, * *p* < .05; n.s. = non-significant.

In the following, we focus on the behavioral results and associated ERPs collected in the rating task. A graphical overview of these results can be found in Figs. 2, 3 and 4. Full linear mixed model outputs of the behavioral and ERP analyses in the rating task are provided in Supplement S3. Supplementary results for the viewing task are provided in Supplement S4.

### Behavioral results

An illustration of rating and response time results can be found in Fig. 2. An alternative illustration showing by-participant differences between Real and Fake conditions per Emotion is provided in Supplement Fig. S5.

### Facial expression ratings

Linear mixed model analysis of ratings during the facial expression task yielded a significant main effect of Information (*b* = −0.98, *t*(30.56) = −3.81, *p* < 0.001), with ratings being more negative in the Fake (*M* = 49.80, 95% CI = [48.97; 50.63]) than the Real condition (*M* = 50.78, 95% CI = [49.93; 51.62]). There were significant effects for both the Negative and the Positive Emotion effect: Rating values for faces showing a neutral facial expression were more positive (*M* = 47.73, 95% CI = [47.40; 48.06]) than those for faces showing an angry expression (*M* = 29.10, 95% CI = [28.55; 29.65]; *b* = 18.63, *t*(34.88) = 12.15, *p* < 0.001), and ratings for faces showing a happy expression were more positive (*M* = 74.04, 95% CI = [73.47; 74.61]) than those for faces with a neutral expression (*b* = 26.32, *t*(31.21) = 12.96, *p* < 0.001). Furthermore, while the interaction of Information and Negative Emotion did not yield a significant result (*b* = −0.14, t(482.02) = −0.31, *p* = 0.758), there was a significant interaction of Information and Positive Emotion (*b* = −1.24, *t*(302.18) = −2.69, *p* = 0.007). Emmeans contrasts revealed a significant Positive Emotion effect (Happy-Neutral) in both the Real (*b* = 26.94, *t*(32.25) = 13.15, *p* < 0.001) and Fake (*b* = 25.70, *t*(31.78) = 12.59, *p* < 0.001) condition. A direct comparison of these effects showed that the effect was significantly larger in the Real as compared to the Fake condition (linear hypothesis test, χ2 = 7.25, *p* = 0.007).

Additionally, the effect of Information was also investigated within each Emotion condition. While ratings for the Fake and Real conditions did not differ in faces with neutral (*b* = −0.61, *t*(104) = −1.75, *p* = 0.124) or angry (*b* = −0.48, *t*(107) =  −1.24, *p* = 0.219) expressions, a significant difference was found in the Happy condition: Faces in the Fake Happy condition received less positive expression ratings (*M* = 73.12, 95% CI = [72.40; 73.84]) than faces in the Real Happy condition (*M* = 74.97, 95% CI = [74.25; 75.69]; *b* = −1.85, *t*(103) = −4.79, *p* < 0.001).

### Response times

Exploratory linear mixed model analysis of response times during the facial expression task yielded no significant effect of Information (*b* = 0.02, *t*(10,350.00) = 1.91, *p* = 0.056). There were significant effects for both the Negative and the Positive Emotion effect: Ratings for faces with neutral expressions were given faster (*M* = 1245.91 ms, 95% CI = [1212.46; 1279.36]) than those for faces showing an angry expression (*M* = 1345.62 ms, 95% CI = [1315.52; 1375.72]; *b* = −0.16, *t*(10,350.03) = −14.44, *p* < 0.001) and also faster than for those showing a happy expression (*M* = 1328.21 ms, 95% CI = [1300.41; 1356.02]; *b* = −0.16, *t*(10,350.03) = −13.68, *p* < 0.001). There were no significant interactions of Information with Negative Emotion (*b* = 0.03, *t*(10,350.00) = 1.21, *p* = 0.228) or with Positive Emotion (*b* = 0.03, *t*(10,350.00) = 1.11, *p* = 0.266).

Investigating the effect of Information within each Emotion condition yielded no differences between the Real and Fake conditions for faces with neutral (*b* = 0.02, *t*(10,350) = 1.14, *p* = 0.382) or angry expressions (*b* = −0.01, *t*(10,350) = −0.56, *p* = 0.574). In the Happy condition, however, a significant difference was found (*b* = 0.04, *t*(10,350) = 2.74, *p* = 0.019). Participants took longer to rate the expression of Fake Happy faces (*M* = 1366.48 ms, 95% CI = [1329.20; 1403.76]) than for that for Real Happy faces (*M* = 1289.88 ms, 95% CI = [1257.04; 1322.71]).

## EEG results

### P1 component

In the linear mixed model analysis, a significant effect of Positive Emotion was found (*b* = −0.46, *t*(10,250.05) = −3.31, *p* < 0.001): The mean P1 amplitude was more negative for faces showing a happy (*M* = 3.54 µV, 95% CI = [3.30; 3.77]) as compared to a neutral expression (*M* = 3.97 µV, 95% CI = [3.73; 4.20]). No other effects reached statistical significance (all *p*s ≥ 0.568).

Planned contrasts for investigating Emotion effects within each Information condition revealed a significant Positive Emotion effect (happy-neutral) in the Real condition (*b* = −0.51, *t*(10,250) = −2.61, *p* = 0.037), with a more negative mean P1 amplitude for Real Happy (*M* = 3.50 µV, 95% CI = [3.20; 3.79]) as compared to Real Neutral faces (*M* = 3.96 µV, 95% CI = [3.67; 4.26]), whereas no significant difference was observed in the Fake condition (*b* = −0.40, *t*(10,250) = −2.07, *p* = 0.077). There was no significant Negative Emotion effect (angry-neutral) in either the Real (*b* = −0.08, *t*(10,250) = −0.38, *p* = 0.845) or Fake condition (*b* = −0.04, *t*(10,250) = −0.20, *p* = 0.845).

Investigating the effects of Information separately within each Emotion condition (Angry, Neutral, Happy) yielded no significant effects (all *p*s ≥ 0.932).

### N170 component

In the linear mixed model analysis, significant effects of Negative Emotion (*b* = 0.48, *t*(28.04) = 3.87, *p* < 0.001) and Positive Emotion were found (*b* = −0.33, *t*(28.71) = −2.75, *p* = 0.010): The mean N170 amplitude was more negative for faces showing an angry (*M* = −2.67 µV, 95% CI = [−2.85; −2.48]) as compared to a neutral expression (*M* = −2.20 µV, 95% CI = [−2.38; −2.02]), and also more negative for faces showing a happy (*M* = −2.53 µV, 95% CI = [−2.72; −2.34]) as compared to a neutral expression. No other effects reached statistical significance (all *p*s ≥ 0.121).

Planned contrasts for investigating Emotion effects within each Information condition revealed a significant Positive Emotion effect (happy-neutral) in the Real condition (*b* = −0.49, *t*(96.1) = −3.07, *p* = 0.006), with a more negative mean N170 amplitude for Real Happy (*M* = −2.56 µV, 95% CI = [−2.80; −2.33]) as compared to Real Neutral faces (*M* = −2.08 µV, 95% CI = [−2.30; −1.85]), whereas no significant difference was observed in the Fake condition (*b* = −0.16, *t*(95.1) = −0.99, *p* = 0.323). The Negative Emotion effect (angry-neutral) was significant in both the Real (*b* = −0.59, *t*(85.8) = −3.57, *p* = 0.002) and Fake conditions (*b* = −0.37, *t*(86.1) = −2.28, *p* = 0.034), with a more negative mean N170 amplitude for Angry as compared to Neutral faces (Real Angry: *M* = −2.67 µV, 95% CI = [−2.90; −2.43]; Real Neutral: *M* = −2.08 µV, 95% CI = [−2.30; −1.85]; Fake Angry: *M* = −2.67 µV, 95% CI = [−2.90; −2.43]; Fake Neutral: *M* = −2.32 µV, 95% CI = [−2.56; −2.09]).

Investigating the effects of Information separately within each Emotion condition (Angry, Neutral, Happy) yielded no significant effects (all *p*s ≥ 0.328).

### EPN

In the linear mixed model analysis, significant effects of Negative Emotion (*b* = 0.63, *t*(30.96) = 4.56, *p* < 0.001) and Positive Emotion were found (*b* = −0.41, *t*(128.61) = −3.76, *p* < 0.001): The mean EPN amplitude was more negative for faces showing an angry (*M* = 0.66 µV, 95% CI = [0.48; 0.83]) as compared to a neutral expression (*M* = 1.28 µV, 95% CI = [1.11; 1.45]), and also more negative for faces showing a happy (*M* = 0.87 µV, 95% CI = [0.69; 1.05]) as compared to a neutral expression. No other effects reached statistical significance (all *p*s ≥ 0.201).

Planned contrasts for investigating Emotion effects within each Information condition revealed a significant positive Emotion effect (happy-neutral) in the Real condition (*b* = −0.54, *t*(455.6) = −3.60, *p* < 0.001), with a more negative mean EPN amplitude for Real Happy (*M* = 0.86 µV, 95% CI = [0.63; 1.08]) as compared to Real Neutral faces (*M* = 1.40 µV, 95% CI = [1.18; 1.61]), whereas no significant difference was observed in the Fake condition (*b* = −0.28, *t*(451.2) = −1.84, *p* = 0.066). The negative Emotion effect (angry-neutral) was significant in both the Real (*b* = −0.69, *t*(75.4) = −4.00, *p* < 0.001) and Fake conditions (*b* = −0.57, *t*(75.6) = −3.29, *p* = 0.002), with a more negative mean EPN amplitude for Angry as compared to Neutral faces (Real Angry: *M* = 0.70 µV, 95% CI = [0.47; 0.93]; Real Neutral: *M* = 1.40 µV, 95% CI = [1.18; 1.61]; Fake Angry: *M* = 0.61 µV, 95% CI = [0.40; 0.83]; Fake Neutral: *M* = 1.16 µV, 95% CI = [0.94; 1.39]).

Investigating the effects of Information separately within each Emotion condition (Angry, Neutral, Happy) yielded no significant effects (all *p*s ≥ 0.366).

### LPP

In the linear mixed model analysis, significant effects of Negative Emotion (*b* = −0.69, *t*(40.87) = −4.71, *p* < 0.001) and Positive Emotion were found (*b* = 0.47, *t*(34.81) = 3.29, *p* = 0.002): The mean LPP amplitude was more positive for faces showing an angry (*M* = 3.24 µV, 95% CI = [3.05; 3.43]) as compared to a neutral expression (*M* = 2.55 µV, 95% CI = [2.37; 2.73]), and also more positive for faces showing a happy (*M* = 3.03 µV, 95% CI = [2.84; 3.22]) as compared to a neutral expression. No other effects reached statistical significance (all *p*s ≥ 0.446).

Planned contrasts for investigating Emotion effects within each Information condition revealed a significant positive Emotion effect (happy-neutral) in the Fake condition (*b* = 0.53, *t*(67.9) = 3.06, *p* = 0.006), with a more positive mean LPP amplitude for Fake Happy (*M* = 3.03 µV, 95% CI = [2.78; 3.27]) as compared to Fake Neutral faces (*M* = 2.50 µV, 95% CI = [2.27; 2.72]), whereas no significant difference was observed in the Real condition (*b* = 0.42, *t*(30.4) = 1.89, *p* = 0.068). The negative Emotion effect (angry-neutral) was significant in both the Real (*b* = 0.59, *t*(32.6) = 2.77, *p* = 0.012) and Fake conditions (*b* = 0.78, *t*(142.9) = 4.69, *p* < 0.001), with a more positive mean LPP amplitude for Angry as compared to Neutral faces (Real Angry: *M* = 3.21 µV, 95% CI = [2.97; 3.46]; Real Neutral: *M* = 2.60 µV, 95% CI = [2.37; 2.83]; Fake Angry: *M* = 3.26 µV, 95% CI = [3.03; 3.50]; Fake Neutral: *M* = 2.50 µV, 95% CI = [2.27; 2.72]).

Investigating the effects of Information separately within each Emotion condition (Angry, Neutral, Happy) yielded no significant effects (all *p*s ≥ 0.964).

## Discussion

AI-generated faces are becoming increasingly prevalent and, at the same time, increasingly difficult to recognize on the basis of purely visual information. In addition to the continued development of deepfake detection techniques^50,51^, dealing with deepfakes as a society requires a more detailed understanding of their psychological and neural impact on perceivers^5^. How effective do images remain even after we know or suspect that the depicted person is not real?

This study investigated how emotional expressions are received when people think that they are or that they are not dealing with real persons. Behavioral expression ratings and brain responses recorded with EEG revealed a pattern of valence-dependent influences of the mere belief of perceiving deepfakes on different stages along the processing stream, from basic- and high-level visual perception to reflexive emotional responses and later evaluations. In a nutshell, while believing a face to be real or fake made no difference for the processing of negative facial expressions, deepfake smiles seem to matter less. These findings will be discussed in detail below.

For angry real faces, we found the typical pattern of more negative expression ratings compared to faces displaying neutral expressions and ERP amplitude modulations in the N170, EPN and LPP components, replicating previous reports^52,53^. We observed comparable emotion effects for presumed deepfakes in all behavioral and ERP measures, suggesting that angry deepfakes have a similarly strong impact as angry real faces.

For smiling faces, we found a different pattern. Happy versus neutral real faces were rated as more positive and were associated with enhanced P1, N170 and EPN amplitudes, replicating previous findings^54,55^. In contrast, happy versus neutral expressions in the deepfake condition were evaluated as more similar compared to their presumed real counterparts in the expression rating and did not differ in the P1, N170 and EPN components. This suggests that smiles actually look different to us when we assume that they come from deepfakes, in the sense that they are less distinct from neutral expressions and that they are emotionally less relevant and arousing. These reduced behavioral and electrophysiological responses indicate that presuming smiles to be deepfake smiles dampens their perceptual and emotional impact.

The LPP component associated with stimulus evaluation showed an enhanced (more positive) amplitude in response to happy versus neutral faces only when the faces were presumed deepfakes, but not when they were taken to be real. This may indicate enhanced evaluative processing demands or enhanced effort invested in evaluation when confronted with fake smiles versus neutral expressions: Our brain may re-evaluate—it may pause and look twice when judging fake smiles. This was corroborated on the behavioral level by reduced positive expression evaluations and slower responses.

The robust impact of angry expressions found irrespective of the presumed realness of the person is in line with other reports of a dominant processing of negative emotional stimuli, suggesting that angry expressions as signals of potential threat are processed with priority and relatively unaffected by context and background information, whereas smiles, like other positive emotional stimuli, are more readily put into perspective based on other available sources of information^10,11,18,30^. We are on guard against angry-looking agents who might harm us, whether they are real people or artificial agents. But a smile matters less when the person we are confronted with actually—or presumably—doesn’t exist.

The valence-dependent pattern speaks against a general account of the present findings as a result of priming induced by the information presented before the faces (real vs. fake). Such an effect should be observed in both conditions, independently of the valence displayed in the facial expressions. Likewise, because, across participants, identical faces were presented in the deepfake and real face condition, physical differences between stimuli were eliminated and cannot explain the modulations of perception-related P1, N170, and EPN effects observed here. In addition, we used cropping masks around the faces (please see Fig. 1) to prevent differential effects of strategic eye movements to the hair or ear regions that are known to sometimes not be ideally represented in deepfakes.

## Limitations and future directions

One limitation of the present study is that an interaction between emotion (happy versus neutral) and information (fake versus real) was observed behaviorally, but not in ERPs. In planned (and pre-registered) separate analyses, the different ERPs show typical effects of happy versus neutral expressions only in the real, but not in the fake condition (P1, N170, and EPN) or vice versa (LPP), but the non-significant interactions weaken conclusions regarding the electrophysiological correlates.

A likely reason is that the displayed emotional expressions were intense and unambiguous, providing strong visual evidence compared to the subtle manipulation of the mere belief that the persons are not real. Presumed deepfake smiles remained at least to some degree strong depictions of smiles. Therefore, residual effects of emotional expressions specifically in the perception-related P1, N170, and EPN components may have weakened interactions. Moreover, the effects of information are distributed across these components along the processing stream from low-level and high-level visual perception to reflexive and evaluative emotion processing, and may contribute in combination to the resulting interaction found in the overall behavioral rating. Consequently, based on the expression ratings alone, we can conclude that deep fake smiles matter less. The observation that happy faces in the deepfake condition did not induce the typical emotion effects often reported in perception-related and reflexive emotional brain responses tentatively suggests that the impact of smiling faces is weakened in a distributed manner across the components often associated with emotional responses.

In addition we would like to point out that the information effects may be influenced by differences between individual observers in many ways, for instance, due to prior beliefs and attitudes towards or expertise with AI. Future research may target such individual differences and replicate the present findings with less pronounced emotional expressions to decrease the strong visual evidence. Finally, in the present ERP study we have presented relatively small faces to avoid artifacts due to eye movements. Previous work has shown that eye-movement patterns differ between genuine (“Duchenne”) and faked (“Non-Duchenne”) smiles^56^. In an adaption of the present design with larger face stimuli, scanning patterns measured with eye tracking could be used to investigate whether similar differences are found between presumed fake and real smiling faces, and thereby reveal valuable evidence on the mechanisms of the processing of deepfake faces.

## Conclusions

Taken together, this study provides first neurocognitive evidence that we are differentially vulnerable to presumed deepfakes displaying positive or negative facial expressions. While angry emotions are processed like those in real faces, deepfake smiles appear less distinct from neutral expressions and have a limited impact on reflexive emotional responses, while at later stages they are evaluated more intensely. This insight into the manifestations of deepfakes as social-emotional stimuli in the human mind and brain can help deal with the phenomenon on a social and individual level. For instance, policies aimed at counteracting misinformation should consider that the impact of positive contents may be especially vulnerable to being labeled as fake, whereas fake negative content may still retain its effectiveness even after being exposed. This also highlights that, in many cases, merely detecting and flagging images as fake may not protect against their negative impact. While the present study focused on faces due to their well-studied effects in capturing attention and transporting social and emotional relevance, and the widespread use of GAN-generated faces, similar findings may extend to other domains, such as AI-generated text, visual art, or music.

### Supplementary Information

Supplementary Information.

## Data Availability

The preregistration, data, and analysis code for this study are available under the following links: https://osf.io/xymz8; https://osf.io/7mj8f. The materials are accessible through the cited face databases.
