# Supplementary material for: Deepfake smiles matter less—the psychological and neural impact of presumed AI-generated faces
Source: Sci Rep. 2023 Sep 26;13:16111. doi: 10.1038/s41598-023-42802-x (PMC10522659; doi:10.1038/s41598-023-42802-x)
Supplement: Supplementary file 1 — Supplementary Information. [file 41598_2023_42802_MOESM1_ESM.pdf]

Supplementary Information

Deepfake Smiles Matter Less—The Psychological and Neural Impact of Presumed AI-generated Faces

Anna Eiserbeck[1,2], Martin Maier[1,2], Julia Baum[1], and Rasha Abdel Rahman[1,2]

[1]Department of Psychology, Faculty of Life Sciences, Humboldt-Universität zu Berlin

[2]Cluster of Excellence Science of Intelligence, Technische Universität Berlin

Supplement S1: Video information and script

Video:

Karras, T. [Tero Karras FI]. (2019, March 3). A Style-Based Generator Architecture for Generative Adversarial Networks [Video]. Youtube. https://www.youtube.com/watch?v=kSLJriaOumA

Script read by the experimenter: (English translation of original German script)

[Video: Show image at 0:27 during explanation; do not play yet]

"Before we start with the experiment, I want to give you some information accompanied by a video:

You may have heard of so-called deepfake faces or GAN faces. These are computer-generated faces—that is, images of faces that are artificially created with the help of computer algorithms. The people you see in such images do not exist in real life. The images in the bottom row are examples of such faces.

These computer algorithms have improved more and more over the years. Nowadays, the results are mostly indistinguishable from real photographs to the naked eye.

How exactly the results that are generated by such algorithms look depends—among other things—on which images are used as a basis. These serve as a template for the images that are created. Depending on the quality of these images, the results will look different.

It is possible to create random faces in the same style or selectively transfer specific attributes from existing images. You're about to see that in the video."

[Video: Play video]

[Video: 0:27 – 1:12]

“The image you see on the bottom left is the initial image. One can now take other images (which you can see in the upper row) and thereby selectively create new artificial images (as you can see in the lower row), representing a combination of the lower left and the respective upper images. For example, the gender, age, hair length, or facial expression can be adjusted.”

[Video: 1:12 – 1:40]

“Here, you can see again how different layers can be adjusted. The image on the right is a combination of the images on the left. Depending on which layer is adjusted, coarse details such as gender, age, and expression change or, for example, only finer facial features or the background and style of the image.”

[Video: Stop video]

“The experiment will also be about such artificial faces.“

Supplement S2: Further details on buildmer specifications

For all models, we used the buildmer package (Voeten, 2020) to specify the random effects structure. It implements an automatic procedure to identify the maximal random effects structure with which the model still converges and to perform backward stepwise elimination to avoid overfitting. Following Matuscheck et al. (2017), both steps were performed in a "backward" manner (i.e., starting with the maximal model and reducing it stepwise), and likelihood ratio tests with $\alpha = .20$ were conducted for model comparisons. lmerControl settings were specified to use the BOBYQA optimizer (bound optimization by quadratic approximation) with a set maximum of 200.000 iterations and to turn off (time-consuming) derivative calculation performed after the optimization is finished.

Supplement S3: Linear mixed model outputs - Rating task

Supplementary Table S3.1.1

LMM statistics for prediction of rating value by Information and Emotion

Variable	*b*	*SE*	*t*	*df*	*p*
Intercept	50.29	0.47	107.53	42.78	**<.001*** **
Information (Fake-Real)	-0.98	0.26	-3.81	30.55	**<.001*** **
Negative Emotion (Neutral-Angry)	18.63	1.53	12.15	34.88	**<.001*** **
Positive Emotion (Happy-Neutral)	26.32	2.03	12.96	31.21	**<.001*** **
Information × Negative Emotion	-0.14	0.44	-0.31	482.01	.758
Information × Positive Emotion	-1.24	0.46	-2.69	302.19	**.007** **
Random effects	*Variance*	*SD*			
Participants (Intercept)	5.09	2.26			
Information	0.95	0.97			
Negative Emotion	61.83	7.86			
Positive Emotion	117.23	10.83			
Items (Intercept)	7.50	2.74			
Information	0.95	0.97			
Negative Emotion	44.43	6.67			
Positive Emotion	31.17	5.58			
Information × Negative Emotion	3.28	1.81			
Information × Positive Emotion	6.33	2.52			
Residual	79.62	8.92			

Model formula:

Rating ~ Information + Emotion + Information:Emotion + (Information + Emotion | Participant) + (Information + Emotion + Information:Emotion | Item)

Note. "×" indicates interactions between fixed factors, *** $p < .001$, ** $p < .01$, * $p < .05$.

Supplementary Table S3.1.2

Emotion effects on rating value separately within each Information condition, computed from the model in Table S3.1.1

Contrast	*b*	*CI*	*SE*	*df*	*t*	*p*
Real: Angry-Neutral	-18.7	-22.8; -14.6	1.55	36.0	-12.1	**<.001 *****
Fake: Angry-Neutral	-18.6	-22.6; -14.5	1.55	36.6	-12.0	**<.001 *****
Real: Happy-Neutral	26.9	21.5; 32.4	2.05	32.2	13.2	**<.001 *****
Fake: Happy-Neutral	25.7	20.3; 31.1	2.04	31.8	12.6	**<.001 *****

Note. *** $p < .001$, ** $p < .01$, * $p < .05$.

Supplementary Table S3.1.3

Information effects on rating value separately within each Emotion condition, computed from the model in Table S3.1.1

Contrast	*b*	*CI*	*SE*	*df*	*t*	*p*
Neutral: Fake-Real	-0.61	-1.47; 0.24	0.35	104	-1.75	.124
Angry: Fake-Real	-0.48	-1.42; 0.46	0.39	107	-1.24	.219
Happy: Fake-Real	-1.85	-2.80; -0.91	0.39	103	-4.79	**<.001 *****

Note. *** $p < .001$, ** $p < .01$, * $p < .05$.

Supplementary Table S3.2.1

LMM statistics for prediction of log-transformed response time by Information and Emotion

Variable	*b*	*SE*	*t*	*df*	*p*
Intercept	6.98	0.07	97.45	29.00	**<.001*** **
Information (Fake-Real)	0.02	0.01	1.91	10350.00	.056
Negative Emotion (Neutral-Angry)	-0.16	0.01	-14.44	10350.03	**<.001*** **
Positive Emotion (Happy-Neutral)	0.16	0.01	13.68	10350.03	**<.001*** **
Information × Negative Emotion	0.03	0.02	1.21	10350.00	.228
Information × Positive Emotion	0.03	0.02	1.11	10350.00	.266
Random effects	*Variance*	*SD*			
Participants (Intercept)	0.15	0.39			
Residual	0.22	0.47			
Model formula: Response time ~ Information + Emotion + Information:Emotion + (1 | Participant)					

Note. "×" indicates interactions between fixed factors, *** p < .001, ** p < .01, * p < .05.

Supplementary Table S3.2.2

Emotion effects on log-transformed response time separately within each Information condition, computed from the model in Table S3.2.1

Contrast	*b*	*CI*	*SE*	*df*	*t*	*p*
Real: Angry-Neutral	0.18	0.14; 0.22	0.02	10350	11.06	**<.001 *****
Fake: Angry-Neutral	0.15	0.11; 0.19	0.02	10350	9.36	**<.001 *****
Real: Happy-Neutral	0.14	0.10; 0.18	0.02	10350	8.89	**<.001 *****
Fake: Happy-Neutral	0.17	0.13; 0.21	0.02	10350	10.47	**<.001 *****

Note. *** $p < .001$, ** $p < .01$, * $p < .05$.

Supplementary Table S3.2.3

Information effects on log-transformed response time separately within each Emotion condition, computed from the model in Table S3.2.1

Contrast	*b*	*CI*	*SE*	*df*	*t*	*p*
Neutral: Fake-Real	0.02	-0.02; 0.06	0.02	10350	1.14	.382
Angry: Fake-Real	-0.01	-0.05; 0.03	0.02	10350	-0.56	.574
Happy: Fake-Real	0.04	0.01; 0.08	0.02	10350	2.74	**.019***

Note. *** $p < .001$, ** $p < .01$, * $p < .05$.

Supplementary Table S3.3.1

LMM statistics for prediction of mean P1 amplitude by Information and Emotion

Variable	*b*	*SE*	*t*	*df*	*p*
Intercept	3.78	0.51	7.37	29.0	**< .001*** **
Information (Fake-Real)	0.06	0.11	0.57	10250.05	.568
Negative Emotion (Neutral-Angry)	0.06	0.14	0.41	10250.03	.682
Positive Emotion (Happy-Neutral)	-0.46	0.14	-3.31	10250.05	**< .001*** **
Information × Negative Emotion	-0.04	0.28	-0.13	10250.06	.894
Information × Positive Emotion	0.11	0.28	0.38	10250.06	.700
Random effects	*Variance*	*SD*			
Participants (Intercept)	7.82	2.80			
Residual	32.90	5.74			

Model formula:

P1 ~ Information + Emotion + Information:Emotion + (1 | Participant)

Note. “×” indicates interactions between fixed factors, *** $p < .001$, ** $p < .01$, * $p < .05$.

Supplementary Table S3.3.2

Emotion effects on P1 amplitude separately within each Information condition, computed from the model in Table S3.3.1

Contrast	*b*	*CI*	*SE*	*df*	*t*	*p*
Real: Angry-Neutral	-0.08	-0.56; 0.41	0.20	10250	-0.38	.845
Fake: Angry-Neutral	-0.04	-0.53; 0.45	0.20	10250	-0.20	.845
Real: Happy-Neutral	-0.51	-1.00; -0.02	0.20	10250	-2.61	**.037***
Fake: Happy-Neutral	-0.40	-0.89; 0.08	0.20	10250	-2.07	.077

Note. *** $p < .001$, ** $p < .01$, * $p < .05$.

Supplementary Table S3.3.3

Information effects on P1 amplitude separately within each Emotion condition, computed from the model in Table S3.3.1

Contrast	*b*	*CI*	*SE*	*df*	*t*	*p*
Neutral: Fake-Real	0.02	-0.45; 0.49	0.20	10250	0.09	.932
Angry: Fake-Real	0.05	-0.42; 0.52	0.20	10250	0.27	.932
Happy: Fake-Real	0.12	-0.35; 0.60	0.20	10250	0.63	.932

Note. *** $p < .001$, ** $p < .01$, * $p < .05$.

Supplementary Table S3.4.1

LMM statistics for prediction of mean N170 amplitude by Information and Emotion

Variable	*b*	*SE*	*t*	*df*	*p*
Intercept	-2.48	0.53	-4.68	29.12	**<.001*** **
Information (Fake-Real)	-0.06	0.09	-0.70	10033.98	.483
Negative Emotion (Neutral-Angry)	0.48	0.12	3.87	28.04	**<.001*** **
Positive Emotion (Happy-Neutral)	-0.33	0.12	-2.75	28.71	**.010***
Information × Negative Emotion	-0.21	0.22	-0.99	10033.30	.324
Information × Positive Emotion	0.33	0.22	1.55	10033.37	.121
Random effects	*Variance*	*SD*			
Participants (Intercept)	8.38	2.89			
Negative Emotion	0.11	0.34			
Positive Emotion	0.07	0.27			
Items (Intercept)	0.10	0.32			
Residual	19.90	4.46			

Model formula:

N170 ~ Information + Emotion + Information:Emotion + (1 + Emotion | Participant) + (1 | Item)

Note. “×” indicates interactions between fixed factors, *** p < .001, ** p < .01, * p < .05.

Supplementary Table S3.4.2

Emotion effects on N170 amplitude separately within each Information condition, computed from the model in Table S3.4.1

Contrast	*b*	*CI*	*SE*	*df*	*t*	*p*
Real: Angry-Neutral	-0.59	-1.01; -0.17	0.16	85.8	-3.57	**.002****
Fake: Angry-Neutral	-0.37	-0.79; 0.05	0.16	86.1	-2.28	**.034***
Real: Happy-Neutral	-0.49	-0.90; -0.08	0.16	96.1	-3.07	**.006****
Fake: Happy-Neutral	-0.16	-0.57; 0.25	0.16	95.1	-0.99	.323

Note. *** $p < .001$, ** $p < .01$, * $p < .05$.

Supplementary Table S3.4.3

Information effects on N170 amplitude separately within each Emotion condition, computed from the model in Table S3.4.1

Contrast	*b*	*CI*	*SE*	*df*	*t*	*p*
Neutral: Fake-Real	-0.24	-0.61; 0.12	0.15	10033	-1.60	.328
Angry: Fake-Real	-0.03	-0.40; 0.33	0.15	10035	-0.21	.836
Happy: Fake-Real	0.09	-0.28; 0.46	0.15	10034	0.60	.833

Supplementary Table S3.5.1

LMM statistics for prediction of mean EPN amplitude by Information and Emotion

Variable	*b*	*SE*	*t*	*df*	*p*
Intercept	0.97	0.58	1.67	29.11	.107
Information (Fake-Real)	-0.10	0.08	-1.15	10055.69	.249
Negative Emotion (Neutral-Angry)	0.63	0.14	4.56	30.96	**<.001*** **
Positive Emotion (Happy-Neutral)	-0.41	0.11	-3.76	128.61	**<.001*** **
Information × Negative Emotion	-0.12	0.21	-0.59	10055.82	.557
Information × Positive Emotion	0.26	0.21	1.28	10056.01	.201
Random effects	*Variance*	*SD*			
Participants (Intercept)	10.14	3.19			
Negative Emotion	0.25	0.50			
Positive Emotion	0.03	0.18			
Items (Intercept)	0.12	0.35			
Residual	18.36	4.29			

Model formula:
EPN ~ Information + Emotion + Information:Emotion + (1 + Emotion | Participant) + (1 | Item)

Note. "×" indicates interactions between fixed factors, *** p < .001, ** p < .01, * p < .05.

Supplementary Table S3.5.2

Emotion effects on EPN amplitude separately within each Information condition, computed from the model in Table S3.5.1

Contrast	*b*	*CI*	*SE*	*df*	*t*	*p*
Real: Angry-Neutral	-0.69	-1.13; -0.25	0.17	75.4	-4.00	**<.001 *****
Fake: Angry-Neutral	-0.57	-1.01; -0.13	0.17	75.6	-3.29	**.002 ****
Real: Happy-Neutral	-0.54	-0.92; -0.16	0.15	455.6	-3.60	**<.001 *****
Fake: Happy-Neutral	-0.28	-0.65; 0.10	0.15	451.2	-1.84	.066

Note. *** $p < .001$, ** $p < .01$, * $p < .05$.

Supplementary Table S3.5.3

Information effects on EPN amplitude separately within each Emotion condition, computed from the model in Table S3.5.1

Contrast	*b*	*CI*	*SE*	*df*	*t*	*p*
Neutral: Fake-Real	-0.23	-0.58; 0.12	0.15	10054	-1.55	.366
Angry: Fake-Real	-0.10	-0.46; 0.25	0.15	10059	-0.72	.712
Happy: Fake-Real	0.04	-0.31; 0.40	0.15	10056	0.26	.792

Supplementary Table S3.6.1

LMM statistics for prediction of mean LPP amplitude by Information and Emotion

Variable	*b*	*SE*	*t*	*df*	*p*
Intercept	2.90	0.39	7.44	29.14	**<.001*** **
Information (Fake-Real)	-0.02	0.10	-0.19	36.21	.849
Negative Emotion (Neutral-Angry)	-0.69	0.15	-4.71	40.87	**<.001*** **
Positive Emotion (Happy-Neutral)	0.47	0.14	3.29	34.81	**.002** **
Information × Negative Emotion	-0.19	0.25	-0.77	51.45	.446
Information × Positive Emotion	0.11	0.27	0.41	38.03	.684
Random effects	*Variance*	*SD*			
Participants (Intercept)	4.49	2.12			
Information	0.08	0.28			
Negative Emotion	0.22	0.47			
Positive Emotion	0.23	0.48			
Information × Negative Emotion	0.44	0.66			
Information × Positive Emotion	0.81	0.90			
Items (Intercept)	0.07	0.27			
Negative Emotion	0.37	0.61			
Positive Emotion	0.21	0.46			
Residual	20.30	4.51			

Model formula:

LPP ~ Information + Emotion + Information:Emotion + (Information + Emotion + Information:Emotion | Participant) + (1 + Emotion | Item)

Note. "×" indicates interactions between fixed factors, *** $p < .001$, ** $p < .01$, * $p < .05$.

Supplementary Table S3.6.2

Emotion effects on LPP amplitude separately within each Information condition, computed from the model in Table S3.6.1

Contrast	*b*	*CI*	*SE*	*df*	*t*	*p*
Real: Angry-Neutral	0.60	0.03; 1.16	0.21	32.6	2.77	**.012***
Fake: Angry-Neutral	0.78	0.36; 1.21	0.17	142.9	4.69	**<.001 *****
Real: Happy-Neutral	0.42	-0.17; 1.01	0.22	30.4	1.89	.068
Fake: Happy-Neutral	0.53	0.09; 0.97	0.17	67.9	3.06	**.006 ****

Note. *** $p < .001$, ** $p < .01$, * $p < .05$.

Supplementary Table S3.6.3

Information effects on LPP amplitude separately within each Emotion condition, computed from the model in Table S3.6.1

Contrast	*b*	*CI*	*SE*	*df*	*t*	*p*
Neutral: Fake-Real	-0.12	-0.57; 0.33	0.18	40.4	-0.67	.964
Angry: Fake-Real	0.07	-0.32; 0.46	0.16	153.1	0.44	.964
Happy: Fake-Real	-0.01	-0.50; 0.48	0.19	31.5	-0.05	.964

Supplement S4: Linear mixed model outputs - Viewing task

Supplementary Table S4.1.1

LMM statistics for prediction of mean P1 amplitude by Information and Emotion

Variable	*b*	*SE*	*t*	*df*	*p*
Intercept	3.89	0.51	7.65	29.2	**< .001*** **
Information (Fake-Real)	-0.20	0.11	-1.78	10144.8	.076
Negative Emotion (Neutral-Angry)	0.06	0.14	0.46	10142.7	.647
Positive Emotion (Happy-Neutral)	-0.43	0.14	-3.10	10142.0	**.002** **
Information × Negative Emotion	0.35	0.27	1.29	10145.0	.199
Information × Positive Emotion	0.14	0.27	0.50	10144.8	.616
Random effects	*Variance*	*SD*			
Participants (Intercept)	7.66	2.77			
Items (Intercept)	0.13	0.36			
Residual	32.45	5.70			

Model formula:

P1 ~ Information + Emotion + Information:Emotion + (1 | Participant) + (1 | Item)

Note. "×" indicates interactions between fixed factors, *** $p < .001$, ** $p < .01$, * $p < .05$.

Supplementary Table S4.1.2

Emotion effects on P1 amplitude separately within each Information condition, computed from the model in Table S4.1.1

Contrast	*b*	*CI*	*SE*	*df*	*t*	*p*
Real: Angry-Neutral	0.11	-0.37; 0.60	0.19	10145	0.58	.559
Fake: Angry-Neutral	-0.24	-0.72; 0.24	0.19	10142	-1.24	.289
Real: Happy-Neutral	-0.50	-0.98; -0.01	0.19	10142	-2.55	**.043***
Fake: Happy-Neutral	-0.36	-0.84; 0.13	0.19	10144	-1.84	.131

Note. *** $p < .001$, ** $p < .01$, * $p < .05$.

Supplementary Table S4.1.3

Information effects on P1 amplitude separately within each Emotion condition, computed from the model in Table S4.1.1

Contrast	*b*	*CI*	*SE*	*df*	*t*	*p*
Neutral: Fake-Real	-0.13	-0.59; 0.34	0.19	10146	-0.66	.767
Angry: Fake-Real	-0.48	-0.94; -0.02	0.19	10144	-2.48	**.040***
Happy: Fake-Real	0.01	-0.46; 0.48	0.19	10144	0.05	.957

Note. *** $p < .001$, ** $p < .01$, * $p < .05$.

Supplementary Table S4.2.1

LMM statistics for prediction of mean N170 amplitude by Information and Emotion

Variable	*b*	*SE*	*t*	*df*	*p*
Intercept	-1.76	0.52	-3.40	29.1	**.002****
Information (Fake-Real)	-0.05	0.09	-0.59	10086.4	.554
Negative Emotion (Neutral-Angry)	0.63	0.16	3.84	27.9	**< .001*****
Positive Emotion (Happy-Neutral)	-0.37	0.12	-3.25	28.5	**.003****
Information × Negative Emotion	0.24	0.22	1.09	10086.3	.277
Information × Positive Emotion	-0.15	0.22	-0.68	10087.5	.497
Random effects	*Variance*	*SD*			
Participants (Intercept)	8.00	2.83			
Negative Emotion	0.56	0.68			
Positive Emotion	0.05	0.21			
Items (Intercept)	0.07	0.26			
Residual	20.27	4.50			

Model formula:
N170 ~ Information + Emotion + Information:Emotion + (1 + Emotion | Participant) + (1 | Item)

Note. "×" indicates interactions between fixed factors, *** $p < .001$, ** $p < .01$, * $p < .05$.

Supplementary Table S4.2.2

Emotion effects on N170 amplitude separately within each Information condition, computed from the model in Table S4.2.1

Contrast	*b*	*CI*	*SE*	*df*	*t*	*p*
Real: Angry-Neutral	-0.51	-1.02; -0.01	0.20	57.7	-2.61	**.015***
Fake: Angry-Neutral	-0.75	-1.26; -0.24	0.20	57.2	-3.81	**.001****
Real: Happy-Neutral	-0.30	-0.70; 0.10	0.16	101.5	-1.90	.060
Fake: Happy-Neutral	-0.45	-0.85; -0.05	0.16	101.1	-2.83	**.011***

Note. *** $p < .001$, ** $p < .01$, * $p < .05$.

Supplementary Table S4.2.3

Information effects on N170 amplitude separately within each Emotion condition, computed from the model in Table S4.2.1

Contrast	*b*	*CI*	*SE*	*df*	*t*	*p*
Neutral: Fake-Real	0.08	-0.29; 0.44	0.15	10087	0.49	.638
Angry: Fake-Real	-0.16	-0.53; 0.21	0.15	10085	-1.05	.638
Happy: Fake-Real	-0.07	-0.44; 0.30	0.15	10087	-0.47	.638

Supplementary Table S4.3.1

LMM statistics for prediction of mean EPN amplitude by Information and Emotion

Variable	*b*	*SE*	*t*	*df*	*p*
Intercept	2.19	0.50	4.41	29.2	**< .001*** **
Information (Fake-Real)	-0.02	0.09	-0.25	178.3	.802
Negative Emotion (Neutral-Angry)	0.73	0.10	7.03	9978.6	**< .001*** **
Positive Emotion (Happy-Neutral)	-0.34	0.10	-3.27	9977.4	**.001** **
Information × Negative Emotion	0.23	0.21	1.10	9978.2	.27
Information × Positive Emotion	0.15	0.21	0.72	9976.6	.473
Random effects	*Variance*	*SD*			
Participants (Intercept)	7.32	2.71			
Items (Intercept)	0.13	0.36			
Information	0.13	0.35			
Residual	18.44	4.29			

Model formula:

EPN ~ Information + Emotion + Information:Emotion + (1 | Participant) + (1 + Information | Item)

Note. "×" indicates interactions between fixed factors, *** $p < .001$, ** $p < .01$, * $p < .05$.

Supplementary Table S4.3.2

Emotion effects on EPN amplitude separately within each Information condition, computed from the model in Table S4.3.1

Contrast	*b*	*CI*	*SE*	*df*	*t*	*p*
Real: Angry-Neutral	-0.61	-0.98; -0.25	0.15	9984	-4.18	**<.001 *****
Fake: Angry-Neutral	-0.84	-1.21; -0.48	0.15	9972	-5.76	**<.001 *****
Real: Happy-Neutral	-0.41	-0.78; -0.05	0.15	9978	-2.82	**.006 ****
Fake: Happy-Neutral	-0.26	-0.63; 0.10	0.15	9975	-1.81	.071

Note. *** $p < .001$, ** $p < .01$, * $p < .05$.

Supplementary Table S4.3.3

Information effects on EPN amplitude separately within each Emotion condition, computed from the model in Table S4.3.1

Contrast	*b*	*CI*	*SE*	*df*	*t*	*p*
Neutral: Fake-Real	0.004	-0.35; 0.36	0.15	1337	0.03	.977
Angry: Fake-Real	-0.22	-0.58; 0.13	0.15	1333	-1.51	.396
Happy: Fake-Real	0.15	-0.20; 0.51	0.15	1343	1.03	.457

Supplementary Table S4.4.1

LMM statistics for prediction of mean LPP amplitude by Information and Emotion

Variable	*b*	*SE*	*t*	*df*	*p*
Intercept	0.31	0.34	0.93	28.9	.362
Information (Fake-Real)	0.16	0.09	1.91	10309.0	.056
Negative Emotion (Neutral-Angry)	-0.30	0.10	-2.84	10309.0	**.004***
Positive Emotion (Happy-Neutral)	0.25	0.10	2.39	10309.0	**.017***
Information × Negative Emotion	0.02	0.21	0.10	10309.0	.917
Information × Positive Emotion	-0.10	0.21	-0.47	10309.0	.637
Random effects	*Variance*	*SD*			
Participants (Intercept)	3.41	1.85			
Residual	18.84	4.34			

Model formula:

LPP ~ Information + Emotion + Information:Emotion + (1 | Participant)

Note. "×" indicates interactions between fixed factors, *** $p < .001$, ** $p < .01$, * $p < .05$.

Supplementary Table S4.4.2

Emotion effects on LPP amplitude separately within each Information condition, computed from the model in Table S4.4.1

Contrast	*b*	*CI*	*SE*	*df*	*t*	*p*
Real: Angry-Neutral	0.31	-0.06; 0.68	0.15	10309	2.08	.070
Fake: Angry-Neutral	0.29	-0.08; 0.66	0.15	10309	1.94	.070
Real: Happy-Neutral	0.30	-0.07; 0.67	0.15	10309	2.02	.070
Fake: Happy-Neutral	0.20	-0.17; 0.57	0.15	10309	1.36	.174

Supplementary Table S4.4.3

Information effects on LPP amplitude separately within each Emotion condition, computed from the model in Table S4.4.1

Contrast	*b*	*CI*	*SE*	*df*	*t*	*p*
Neutral: Fake-Real	0.20	-0.15; 0.56	0.15	10309	1.38	.328
Angry: Fake-Real	0.18	-0.17; 0.54	0.15	10309	1.23	.328
Happy: Fake-Real	0.11	-0.25; 0.46	0.15	10309	0.71	.478

Supplement S5: Additional Figures

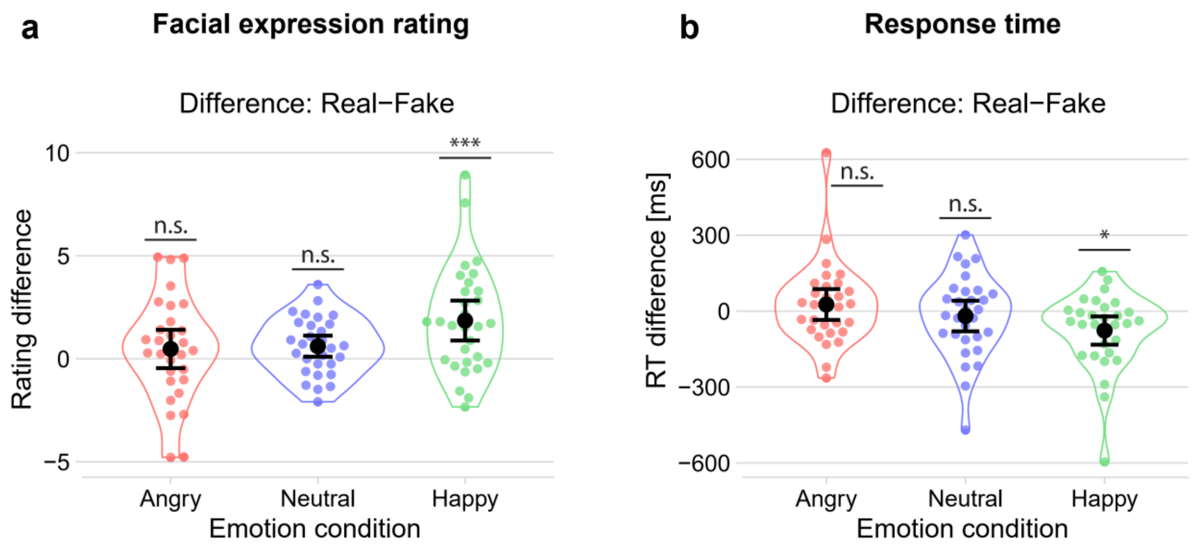

Figure S5. Difference values between Real and Fake condition per Emotion condition in facial expression ratings (a) and response times (b). The colored dots represent by-participant differences of the respective conditions; the black dot represents the mean difference; error bars display 95% confidence intervals around the mean. Asterisks indicates a significant difference between the respective conditions observed in the analyses. *** $p < .001$, ** $p < .01$, * $p < .05$; n.s. = non-significant.
